# Foreword

**Published:** 2009-10

**Authors:** DeLois Greenwood

**Affiliations:** Vice President, The Smile Train, USA E-mail: dgreenwood@smiletrain.org

On behalf of the leadership of The Smile Train, I wish to extend my support to the Association of Plastic Surgeons of India and to this special issue of the Indian Journal of Plastic Surgery, which is dedicated to cleft lip and palate. We all are partners in the mission of the Smile Train, an organization established 10 years ago, that no child should be forced to live a life of shame and isolation because of a cleft.

The members of The Association of Plastic Surgeons of India have demonstrated a passionate quest to deliver the highest quality of comprehensive cleft care, and the pioneering work in this supplement will continue to further this goal. The vast body of knowledge contained herein will surely benefit and inspire the next generation of skilled cleft surgeons.

Cleft lip and palate is one of the world's most common birth defects affecting millions of children and their families. Across the globe, cleft patients face challenges, both physical and social. Many families living in poverty are prevented from receiving the care they so desperately need. Providing treatment for every child in need is a big challenge, but a challenge that has been accepted by a dedicated group of like-minded people. Forming strategic alliances, collaborating, sharing resources, talent, experience, and expertise are keys to advancing cleft care. Our support of this supplement and continued partnership with the IJPS is an example of how, by working together, we can accomplish things that none of us could do alone.

The Smile train supports over 70 countries and has sponsored over 500 000 patients since inception. In India, the Smile Train supports 165 centers that have performed nearly 200 000 surgeries since the inception of The Smile Train India in 2000. The Smile Train's three priorities are safety, treatment, and training. All the Smile Train Centers are critically evaluated to ensure that adequate facilities and skills are available to ensure safety for children undergoing surgeries. Very carefully designed and highly sophisticated software collects all the records of surgeries and assigns these on a sample basis to a high power panel for critical evaluation. Each surgeon's results are continually monitored, evaluated, measured, and tracked. We strongly support continuing education of all medical professionals associated with clefts. The idea is not just to repair clefts but raise medical standards in the countries we operate in. When the last cleft is operated we want to leave the country's treatment infrastructure in a better position than we found it!

